# Supplementary material for: A Likelihood Approach for Real-Time Calibration of Stochastic Compartmental Epidemic Models
Source: PLoS Comput Biol. 2017 Jan 17;13(1):e1005257. doi: 10.1371/journal.pcbi.1005257 (PMC5240920; doi:10.1371/journal.pcbi.1005257)
Supplement: S1 File — (TAR.GZ) [file pcbi.1005257.s014.tar.gz › HSPH_Online-SI-Revision/EnKF07/n100-extreme/n100-extreme_EnKF_table-4.pdf]

1	R0	{2.76052, 16.3913}	{2.20157, 8.50332}	{{0.343864, 2.6007}, {3., 13.4414}}	{30.4848, 48.123}	inf	10 419.	3771.67	{110.731, 2762.1}	92.1027	1.0643	newly	5804.	3069.24	{61., 4869.}	95.9793	0.971394	Reff	2.28524	0.426955	{0.0688838, 0.635087}	81.3169	ar	92 090.	176 836.	{167 062., 198 573.}	92.0252
2	R0	{2.18202, 15.1734}	{2.70032, 4.26755}	{{2.14661, 1.25253}, {3., 6.30923}}	{24.2267, 71.8748}	inf	10 970.	4350.79	{948.674, 8011.36}	84.6603	0.683501	newly	5914.	8990.85	{1420., 8761.}	155.594	0.470487	Reff	1.6841	0.577775	{0.137952, 0.767897}	70.2133	ar	84 264.	184 384.	{169 619., 196 520.}	118.817
3	R0	{2.57891, 12.5971}	{2.49055, 6.64515}	{{1.42371, 3.24038}, {3., 10.0481}}	{22.0405, 47.2486}	inf	8525.	1770.99	{62.8291, 2068.01}	83.0736	1.09025	newly	6571.	2669.76	{29., 2277.}	95.4795	1.09497	Reff	2.202	0.45414	{0.0236145, 0.743427}	79.376	ar	90 302.	178 011.	{167 042., 201 407.}	97.1289
4	R0	{2.5856, 9.28508}	{2.45971, 4.70317}	{{1.76624, 1.19639}, {3., 12.7463}}	{20.5757, 65.6951}	inf	2564.	21 306.5	{4143.9, 54 072.9}	736.394	0.776827	newly	3492.	26 712.5	{2326., 72 811.}	676.767	0.717474	Reff	2.47933	0.638297	{0.168819, 1.36158}	74.2553	ar	90 312.	154 786.	{93 968., 247 921.}	75.5808
5	R0	{2.4406, 12.1341}	{2.50783, 4.53576}	{{2.00105, 1.18596}, {3., 7.46776}}	{21.0827, 62.6197}	inf	4472.	1941.58	{744.043, 2289.47}	79.3437	0.578608	newly	3644.	3395.44	{681., 4685.}	99.2514	0.496843	Reff	2.2517	0.568662	{0.169282, 1.92837}	74.7452	ar	88 597.	182 378.	{166 372., 229 198.}	105.851
6	R0	{2.72155, 15.4094}	{2.20881, 8.55373}	{{0.358459, 2.64231}, {3., 13.5196}}	{30.9129, 44.4902}	inf	8392.	4112.17	{79.6577, 2776.03}	89.7905	0.867579	newly	5118.	3325.04	{65., 4346.}	92.7737	0.81733	Reff	2.34317	0.422081	{0.0701384, 0.638307}	81.9868	ar	91 682.	177 139.	{166 538., 199 805.}	93.2102
7	R0	{2.63781, 14.3935}	{2.2087, 5.59188}	{{0.116137, 3.46397}, {3., 12.5197}}	{32.4698, 61.15}	inf	9079.	7151.67	{143.87, 4326.49}	114.483	0.822132	newly	5875.	5523.09	{161., 6404.}	106.361	0.653455	Reff	2.22919	0.488573	{0.0308464, 0.713445}	78.0829	ar	91 192.	181 875.	{164 529., 178 102.}	99.442
8	R0	{2.82391, 16.104}	{2.2094, 8.55552}	{{0.360696, 2.63508}, {3., 13.5263}}	{29.1192, 46.8733}	inf	8948.	4105.69	{81.3351, 2768.34}	89.5273	0.889801	newly	5170.	3314.65	{76., 4166.}	92.8551	0.817625	Reff	2.41478	0.421941	{0.0707605, 0.636952}	82.5267	ar	92 664.	177 122.	{166 615., 198 313.}	91.1441
9	R0	{2.80645, 14.6457}	{2.19324, 8.61462}	{{0.38132, 2.67316}, {3., 13.6196}}	{29.8502, 41.1799}	inf	5809.	4449.08	{249.625, 2789.43}	86.3591	0.536171	newly	4055.	3684.49	{109., 4269.}	87.0557	0.541977	Reff	2.54301	0.406413	{0.0709835, 0.651865}	84.0184	ar	92 567.	177 545.	{168 217., 196 597.}	91.8021
10	R0	{1.86213, 5.4271}	{2.15638, 4.12358}	{{1.05384, 2.6471}, {3., 4.98071}}	{46.2517, 29.8597}	inf	1977.	15 473.9	{5130.09, 48 935.5}	687.694	0.769217	newly	4393.	18 708.3	{5350., 56 277.}	333.24	0.480455	Reff	1.77612	0.433099	{0.0442804, 1.0393}	76.3453	ar	75 362.	167 455.	{98 096., 240 715.}	123.671
11	R0	{2.27349, 14.6338}	{2.60152, 5.54591}	{{1.62041, 3.48145}, {3., 12.4007}}	{28.2068, 62.1021}	inf	10 491.	6421.81	{275.412, 4571.62}	104.283	0.818474	newly	6044.	6064.34	{412., 6179.}	108.499	0.598478	Reff	1.80677	0.621214	{0.127657, 0.76139}	68.9083	ar	85 805.	182 174.	{162 783., 203 306.}	112.311
12	R0	{2.77261, 14.1894}	{2.5551, 12.3823}	{{1.34384, 4.87748}, {3., 18.6763}}	{17.9465, 40.9943}	inf	6492.	2584.75	{389.172, 2777.15}	75.3795	0.613529	newly	4508.	2595.09	{345., 1905.}	103.975	0.748785	Reff	2.48165	0.548283	{0.226218, 1.22555}	77.9065	ar	92 508.	178 124.	{166 777., 196 954.}	92.5503
13	R0	{2.65402, 19.3935}	{2.37981, 6.45327}	{{0.91826, 0.672472}, {3., 19.448}}	{26.2359, 66.869}	inf	11 250.	4844.9	{987.02, 5778.96}	59.1319	0.482694	newly	4926.	7973.02	{873., 11 754.}	121.361	0.461333	Reff	2.14402	0.503303	{0.133038, 0.687672}	76.9231	ar	91 184.	181 888.	{167 194., 226 282.}	99.4736
14	R0	{1.75297, 4.45354}	{2.48776, 4.71598}	{{2.00481, 1.26725}, {3., 7.53103}}	{43.8448, 50.7322}	inf	699.	2314.61	{758.187, 2263.42}	237.766	0.313463	newly	1855.	3553.57	{679., 4797.}	159.965	0.406625	Reff	1.72278	0.622618	{0.174661, 2.00479}	68.0663	ar	71 263.	184 174.	{166 025., 248 604.}	160.322
15	R0	{1.99928, 7.1501}	{2.46793, 4.36785}	{{2.00047, 1.15227}, {3., 7.46333}}	{30.7115, 42.1143}	inf	3389.	1948.39	{736.711, 2783.62}	75.8208	0.47177	newly	4922.	3541.71	{689., 6645.}	92.8901	0.5844	Reff	1.85853	0.571105	{0.169159, 1.93067}	70.8761	ar	79 602.	183 383.	{166 731., 229 368.}	130.375
16	R0	{2.67398, 10.3134}	{2.51911, 4.48811}	{{2.00285, 1.2109}, {3., 7.48603}}	{18.7157, 56.4827}	inf	4133.	1947.85	{753.925, 2288.44}	78.2998	0.54539	newly	4823.	3395.24	{688., 4948.}	91.1089	0.573341	Reff	2.49563	0.569663	{0.166625, 1.94933}	77.1735	ar	91 289.	182 259.	{166 241., 229 587.}	99.6511
17	R0	{2.44634, 16.3226}	{2.36439, 6.61497}	{{0.910681, 0.690437}, {3., 19.4421}}	{30.9614, 69.7858}	inf	10 530.	4687.63	{913.066, 5789.13}	58.3725	0.509792	newly	5419.	7601.88	{987., 11 442.}	108.407	0.493156	Reff	1.9738	0.511836	{0.131412, 0.682241}	75.0221	ar	88 710.	181 899.	{167 480., 226 623.}	105.049
18	R0	{2.02168, 10.1676}	{2.23833, 5.61209}	{{0.125356, 3.4635}, {3., 12.5279}}	{48.3213, 53.6254}	inf	4597.	6912.6	{353.712, 4280.02}	112.119	0.41138	newly	4153.	5793.3	{368., 6284.}	108.013	0.442966	Reff	1.82331	0.494605	{0.0330878, 0.712847}	72.8733	ar	80 299.	181 987.	{167 214., 177 989.}	126.637
19	R0	{2.89969, 15.009}	{2.21833, 8.61051}	{{0.37878, 2.66258}, {3., 13.6158}}	{27.5796, 42.631}	inf	6390.	4400.2	{305.055, 2790.69}	87.0626	0.595438	newly	4254.	3675.54	{132., 4221.}	87.1622	0.582349	Reff	2.60468	0.409354	{0.0711143, 0.652313}	84.2839	ar	93 349.	177 604.	{168 414., 195 765.}	90.2576
20	R0	{2.68933, 7.34928}	{2.38515, 4.94802}	{{1.76348, 1.27715}, {3., 12.7993}}	{22.1694, 64.7548}	inf	478.	22 915.	{4847.24, 53 876.6}	4695.41	1.49615	newly	1059.	28 096.5	{2648., 78 653.}	2556.69	1.20488	Reff	2.66728	0.646935	{0.255522, 1.36517}	75.7455	ar	91 574.	154 595.	{84 923., 248 306.}	74.0018
21	R0	{2.15352, 9.28074}	{2.45869, 4.37324}	{{2.00094, 1.16829}, {3., 7.4628}}	{26.1365, 52.8784}	inf	3961.	1937.85	{744.188, 2794.25}	78.1177	0.53563	newly	4282.	3519.65	{687., 6632.}	95.3865	0.543179	Reff	1.99267	0.567851	{0.169062, 1.93102}	71.5858	ar	83 845.	183 252.	{166 637., 229 279.}	118.56
22	R0	{2.21953, 9.04387}	{1.97313, 4.06967}	{{0.0604808, 0.933991}, {3., 5.80265}}	{44.1552, 57.6109}	inf	3707.	5140.72	{823.881, 17 665.}	89.8183	0.314332	newly	4322.	5953.59	{619., 16 532.}	104.292	0.407039	Reff	2.06581	0.272709	{0.00866617, 0.455334}	86.7989	ar	84 856.	167 030.	{156 583., 205 391.}	103.294
23	R0	{2.21987, 14.5347}	{2.66733, 4.10093}	{{2.15047, 1.283}, {3., 6.31396}}	{21.2895, 71.7852}	inf	10 313.	5080.43	{1101.04, 8435.61}	90.5918	0.684511	newly	5905.	12 243.3	{1385., 12 305.}	215.023	0.533386	Reff	1.76493	0.602688	{0.156106, 1.85269}	71.0158	ar	85 067.	190 857.	{171 611., 263 415.}	124.361
24	R0	{2.84268, 17.9659}	{2.61974, 3.92811}	{{2.17959, 1.36171}, {3., 6.30241}}	{13.0448, 78.1357}	inf	10 277.	6477.08	{692.245, 12 846.5}	96.8177	0.702791	newly	5220.	16 058.7	{1203., 58 258.}	304.334	0.559171	Reff	2.35894	0.657784	{0.163067, 1.93351}	72.664	ar	92 836.	197 953.	{171 511., 287 032.}	113.228
25	R0	{2.52706, 10.3679}	{2.58581, 12.3757}	{{1.37546, 4.89427}, {3., 18.7321}}	{21.6118, 52.9727}	inf	4409.	2515.6	{323.809, 2771.44}	69.7861	0.489896	newly	4655.	2542.72	{277., 1927.}	104.302	0.787024	Reff	2.33579	0.538013	{0.21594, 0.70936}	76.9665	ar	89 623.	177 955.	{166 855., 173 982.}	98.5598
26	R0	{2.62835, 19.1088}	{2.3632, 6.46391}	{{0.904144, 0.699493}, {3., 19.4366}}	{27.3389, 67.0631}	inf	12 549.	4335.53	{161.278, 5773.49}	67.0196	0.691804	newly	5505.	7015.97	{252., 11 444.}	105.508	0.56434	Reff	2.05547	0.511965	{0.129603, 0.676263}	75.7813	ar	90 908.	181 416.	{167 044., 226 754.}	99.5603
27	R0	{2.76831, 19.4155}	{2.59867, 5.52495}	{{1.61536, 3.49325}, {3., 12.3529}}	{16.3384, 71.5436}	inf	16 382.	6439.9	{264.816, 4702.73}	99.2718	1.00149	newly	6900.	6305.3	{300., 6639.}	107.98	0.660976	Reff	1.96727	0.640633	{0.160531, 0.764739}	70.0625	ar	92 175.	182 998.	{163 916., 235 732.}	98.5331
28	R0	{2.96609, 16.8903}	{2.47011, 4.08095}	{{1.99515, 1.04798}, {3., 7.39142}}	{17.8877, 75.8385}	inf	11 248.	1912.3	{683.75, 2688.39}	87.4757	0.976836	newly	6075.	3640.28	{712., 6643.}	90.531	0.670501	Reff	2.44352	0.569828	{0.196024, 1.89167}	76.6801	ar	94 024.	183 506.	{166 941., 228 718.}	95.1689
29	R0	{2.17046, 14.39}	{2.69901, 4.17799}	{{2.15996, 1.3187}, {3., 6.30013}}	{24.5166, 70.966}	inf	8850.	4111.84	{995.192, 8413.37}	83.5067	0.623423	newly	5160.	8635.23	{1375., 10 389.}	169.841	0.471225	Reff	1.78813	0.545522	{0.153777, 0.705218}	72.9663	ar	84 001.	186 139.	{171 308., 198 306.}	121.591
30	R0	{2.88772, 12.4647}	{2.46933, 4.4975}	{{1.81067, 1.15768}, {3., 12.733}}	{18.3769, 65.3267}	inf	3959.	22 241.6	{4231.52, 57 359.8}	471.685	0.637104	newly	3670.	27 721.4	{2492., 72 638.}	667.114	0.716957	Reff	2.71599	0.620316	{0.125892, 1.27407}	77.1606	ar	93 273.	154 947.	{94 171., 247 679.}	70.0589
31	R0	{2.46831, 8.14308}	{2.53179, 7.18192}	{{2.16239, 4.27134}, {3., 14.692}}	{17.6524, 42.1568}	inf	1290.	22 894.8	{6631.09, 42 674.9}	1675.2	1.11331	newly	2181.	24 384.5	{6365., 59 505.}	1020.39	0.893384	Reff	2.41433	0.975985	{0.325149, 2.18626}	59.5753	ar	88 762.	154 079.	{85 624., 224 693.}	78.2727
32	R0	{1.93236, 7.38987}	{1.97251, 4.06531}	{{0.0593116, 0.937439}, {3., 5.79252}}	{54.0583, 49.0751}	inf	2899.	5130.69	{817.187, 17 635.1}	119.799	0.334726	newly	4137.	5940.56	{630., 16 511.}	108.09	0.404349	Reff	1.80908	0.272316	{0.00846765, 0.45548}	84.9472	ar	77 654.	166 773.	{156 387., 205 038.}	120.704
33	R0	{2.82251, 17.6954}	{2.46999, 6.62927}	{{1.43374, 3.28338}, {3., 10.0992}}	{19.4067, 62.5368}	inf	8313.	2090.27	{787.782, 2061.85}	79.2839	0.812491	newly	4307.	3025.24	{471., 2852.}	100.417	0.682794	Reff	2.44362	0.483061	{0.0334124, 0.9505}	80.2317	ar	92 745.	179 239.	{168 250., 213 158.}	93.2599
34	R0	{1.87083, 12.3994}	{2.69572, 4.30489}	{{2.15489, 1.30693}, {3., 6.29794}}	{44.0922, 65.2815}	inf	6523.	4048.08	{1005.56, 7114.97}	87.4395	0.532012	newly	4361.	8963.18	{1392., 7122.}	198.307	0.427157	Reff	1.57679	0.582802	{0.149112, 0.790268}	70.098	ar	76 282.	186 754.	{171 465., 222 471.}	144.821
35	R0	{2.95779, 16.3003}	{2.50256, 6.57015}	{{1.44445, 3.31415}, {3., 10.1729}}	{16.9892, 59.6931}	inf	6128.	2228.51	{1480.15, 2243.05}	75.0265	0.578946	newly	3852.	3134.89	{727., 3297.}	99.561	0.510852	Reff	2.67834	0.478073	{0.0272415, 0.959762}	82.1504	ar	93 873.	179 531.	{168 219., 212 626.}	91.2489
36	R0	{2.18718, 14.261}	{2.54843, 12.135}	{{1.16514, 4.82809}, {3., 18.3365}}	{30.6256, 40.4187}	inf	9128.	3007.06	{1113.32, 2996.75}	76.1585	0.602337	newly	5307.	2790.	{783., 2280.}	96.5676	0.649112	Reff	1.78461	0.562818	{0.229198, 0.745657}	69.8677	ar	84 379.	177 890.	{168 381., 174 793.}	110.822
37	R0	{2.44912, 9.31118}	{2.14474, 4.10127}	{{1.05212, 2.64688}, {3., 4.9865}}	{33.5834, 56.0217}	inf	3260.	15 384.5	{5076.24, 48 612.9}	380.657	0.578605	newly	3995.	18 630.3	{4015., 56 466.}	374.226	0.514627	Reff	2.30876	0.434592	{0.0419887, 1.04441}	81.2764	ar	88 442.	167 728.	{97 971., 240 982.}	92.5709
38	R0	{2.05949, 5.72514}	{2.2573, 4.14946}	{{1.05002, 2.64605}, {3., 5.00319}}	{41.8472, 32.6879}	inf	809.	14 192.6	{4927.38, 27 905.3}	1659.17	1.11619	newly	2064.	16 055.4	{3935., 42 644.}	683.75	0.725352	Reff	2.02491	0.361119	{0.0427068, 0.788403}	82.1662	ar	81 587.	160 180.	{80 957., 207 079.}	101.748
39	R0	{1.75505, 8.52404}	{2.27783, 5.59486}	{{0.0616247, 3.46954}, {3., 12.4764}}	{58.5912, 51.9834}	inf	4468.	6806.71	{486.643, 4319.47}	115.106	0.390859	newly	4543.	6129.22	{445., 6423.}	112.254	0.452306	Reff	1.55847	0.527578	{0.0167636, 0.725343}	67.6163	ar	70 925.	182 568.	{167 301., 178 452.}	157.41
40	R0	{2.24724, 13.7861}	{2.5414, 12.0899}	{{1.12947, 4.81858}, {3., 18.2601}}	{29.2493, 41.2923}	inf	10 101.	2802.38	{93.6713, 3194.56}	79.9072	0.821738	newly	6145.	2663.92	{62., 2427.}	97.3448	0.878377	Reff	1.78404	0.579705	{0.246298, 0.7533}	68.8951	ar	85 257.	178 006.	{167 592., 174 935.}	108.787
41	R0	{1.77635, 8.62406}	{2.19567, 8.45628}	{{0.331891, 2.57016}, {3., 13.3723}}	{62.764, 48.0532}	inf	5330.	3707.05	{148.774, 2695.78}	102.182	0.788142	newly	5334.	3004.53	{83., 4443.}	97.7801	0.909743	Reff	1.53452	0.424845	{0.0669828, 0.636266}	72.444	ar	72 941.	176 339.	{166 992., 197 625.}	141.756
42	R0	{1.80397, 5.4932}	{2.18619, 4.21834}	{{1.05451, 2.64717}, {3., 4.97884}}	{49.3905, 30.2797}	inf	2276.	16 448.3	{5123.4, 50 970.4}	627.948	0.733486	newly	4341.	19 757.8	{5311., 56 506.}	362.298	0.489168	Reff	1.7106	0.408201	{0.0443078, 1.01285}	76.9714	ar	73 232.	166 869.	{98 440., 267 292.}	129.204
43	R0	{2.83096, 16.7077}	{2.32134, 6.81521}	{{0.929386, 0.587856}, {3., 19.4538}}	{24.9283, 68.4101}	inf	8628.	4784.83	{333.536, 6041.93}	49.453	0.453702	newly	4809.	8124.6	{375., 12 455.}	135.606	0.581198	Reff	2.43862	0.498993	{0.1418, 0.634508}	79.5379	ar	92 740.	182 975.	{166 032., 226 560.}	97.2994
44	R0	{2.73678, 11.6021}	{2.00928, 4.08147}	{{0.0518716, 0.933109}, {3., 5.79298}}	{36.3925, 65.972}	inf	6552.	5556.55	{971.339, 17 756.6}	76.0466	0.420067	newly	6185.	6350.38	{827., 16 585.}	84.4783	0.430502	Reff	2.44682	0.279	{0.00298041, 0.453056}	88.5974	ar	92 003.	169 781.	{157 080., 230 452.}	90.1161
45	R0	{2.14235, 6.98601}	{2.50891, 4.48499}	{{2.00242, 1.2025}, {3., 7.48732}}	{26.3947, 41.2199}	inf	2850.	1945.72	{747.913, 2288.28}	73.6654	0.403055	newly	4808.	3400.49	{689., 5113.}	91.1213	0.572751	Reff	2.02463	0.568424	{0.167427, 1.94729}	71.9245	ar	82 974.	182 518.	{166 475., 229 520.}	119.97
46	R0	{2.23409, 9.21289}	{2.52996, 4.61797}	{{2.00249, 1.21729}, {3., 7.4994}}	{23.268, 49.8749}	inf	2544.	2271.7	{732.352, 2283.46}	88.1576	0.39437	newly	3017.	3678.11	{683., 6659.}	117.734	0.470347	Reff	2.12542	0.597792	{0.167442, 2.00247}	71.8742	ar	85 163.	184 547.	{166 372., 247 452.}	116.699
47	R0	{2.12258, 8.15265}	{2.16419, 4.07985}	{{1.05497, 2.64745}, {3., 4.972}}	{39.2318, 51.1683}	inf	2945.	15 394.7	{5039.34, 49 371.2}	430.647	0.612669	newly	3839.	18 685.9	{3972., 56 315.}	393.213	0.527409	Reff	1.99728	0.440523	{0.0460475, 1.04617}	78.3714	ar	82 690.	168 233.	{98 987., 241 180.}	105.53
48	R0	{2.71771, 10.4636}	{2.1789, 4.30776}	{{1.15447, 2.64618}, {3., 5.00041}}	{29.4629, 58.831}	inf	2061.	15 960.4	{8727.99, 47 901.9}	676.796	0.766696	newly	2437.	19 819.	{6635., 56 360.}	715.694	0.707523	Reff	2.62862	0.418253	{0.0406248, 0.961426}	84.0964	ar	91 716.	169 797.	{91 127., 269 322.}	89.2743
49	R0	{2.44369, 6.66477}	{2.53166, 7.07992}	{{2.1628, 4.28076}, {3., 14.6909}}	{17.7754, 43.9713}	inf	1213.	22 425.5	{6631.46, 41 189.5}	1749.02	1.13318	newly	3032.	24 468.5	{6443., 66 570.}	711.434	0.761522	Reff	2.39345	0.96202	{0.324977, 2.18603}	59.8061	ar	88 827.	153 484.	{85 710., 224 713.}	77.4657
50	R0	{2.77383, 17.9775}	{2.31224, 6.87461}	{{0.899217, 0.751029}, {3., 19.4916}}	{26.0993, 66.4709}	inf	14 883.	3961.48	{509.402, 5778.37}	74.8123	0.753321	newly	6948.	6045.96	{699., 11 429.}	88.7254	0.533362	Reff	2.0682	0.512046	{0.126843, 0.671305}	75.8762	ar	92 334.	179 006.	{165 808., 225 148.}	93.8678
